# Molar Incisor Hypomineralization: Etiology, Correlation with Tooth Number Anomalies and Implications for Comprehensive Management Strategies in Children from Transylvania

**DOI:** 10.3390/diagnostics14212370

**Published:** 2024-10-24

**Authors:** Laura-Roxana Contac, Silvia Izabella Pop, Septimiu Voidazan, Cristina Ioana Bica

**Affiliations:** 1 Faculty of Dental Medicine, Pedodontics Department, University of Medicine, Pharmacy, Science and Technology ‘George Emil Palade’ of Târgu Mureş, 540142 Târgu Mureș, Romania; lauracontac92@gmail.com (L.-R.C.);; 2 Faculty of Dental Medicine, Orthodontics Department, University of Medicine, Pharmacy, Science and Technology ‘George Emil Palade’ of Târgu Mureş, 540142 Târgu Mureș, Romania; 3 Epidemiology Department, University of Medicine, Pharmacy, Science and Technology ‘George Emil Palade’ of Târgu Mureş, 540142 Târgu Mureș, Romania

**Keywords:** molar hypomineralization, hypodontia, mixed dentition, dental agenesia, conservative treatment

## Abstract

Background/Objectives: This study investigates the etiology of enamel developmental defects, specifically Molar Incisor Hypomineralization (MIH), and explores correlations between MIH and dental anomalies such as hypodontia to improve interdisciplinary restorative and orthodontic treatments. Additionally, it assesses the influence of stress factors on the development of enamel defects. Methods: Conducted from July to September 2024, this study involved 57 patients aged 6 to 11 from an urban setting, divided into two groups: 32 with MIH and 25 controls, selected based on criteria of mixed dentition without systemic pathology or chronic medication. Clinical evaluations, including intraoral photographs and panoramic radiographs, were performed alongside a detailed questionnaire addressed to the mothers covering prenatal, perinatal, and postnatal factors. Results: The average age of children with MIH was 7.5 years, in contrast to 7.04 years in the control group (*p* = 0.17). Significant differences were noted in maternal age, with MIH mothers older (35.56 years) than controls (29.36, *p* = 0.0001). The prevalence of MIH was higher in boys (66.7%) compared to girls (38.1%, *p* = 0.036). Factors such as prolonged labor, medication during birth, and early postnatal medication were significantly linked to MIH. The study shows a strong correlation with hypodontia, with a significantly higher incidence of tooth number anomalies (*p* = 0.009) in the study group. Conclusions: Overall, the study emphasizes the association of MIH with various maternal and birth-related factors and with hypodontia, highlighting the need for a comprehensive, multidisciplinary approach to diagnosis and treatment. Further research is recommended to investigate the relationship between stress factors and MIH.

## 1. Introduction

Dental development is a complex process regulated by a series of molecular and cellular interactions. Disruptions and alterations in the mechanisms of tooth formation during the initiation, morphogenesis, and histodifferentiation phases can lead to the occurrence of developmental dental anomalies (DDA). DDA presents a wide variety of manifestations and is generally classified into abnormalities of number, size, shape, and structure (Table 1) [1,2].

**Figure 1:**
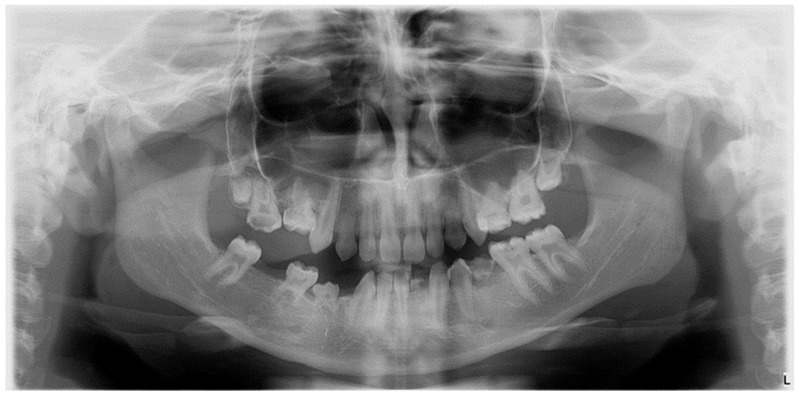
Radiographic image of developmental dental anomalies (number, size, structure).

The etiology of DDA is multifactorial, involving hereditary factors, environmental influences, or combinations of these (Figure 1) [3].

These anomalies can occur isolated or in association with other anomalies and/or syndromes. One of the most frequently encountered developmental anomalies is Molar-incisor hypomineralization (MIH), which leads to both functional and aesthetic disturbances, often requiring dental treatments such as direct restorations, endodontic therapy, tooth extraction, orthodontic management of malocclusion, or complex rehabilitations [4,5]. Various medical conditions are associated with specific types of developmental dental tissue anomalies. The management of patients with enamel development anomalies, such as MIH, often requires a multidisciplinary approach [6].

**Figure 2:**
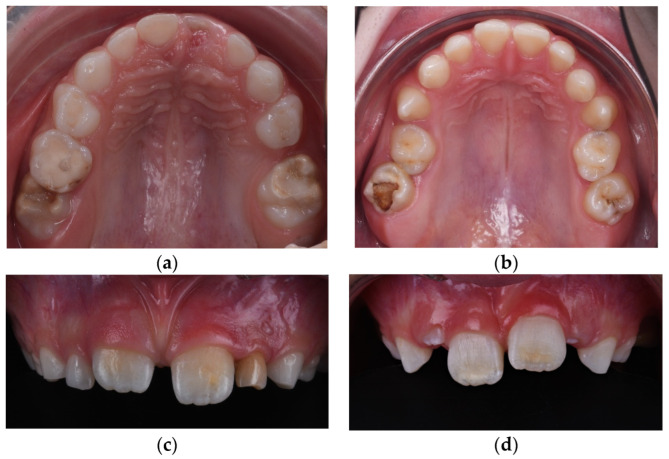
Clinical diagnosis of Molar incisor hypomineralization (MIH) based on visible enamel defects in mixed dentition; (**a**) Impacted first permanent molar with MIH grade 3 and atypical restoration; (**b**) MIH with post-eruptive enamel breakdown (PEB) and Developmental Dental Anomalies; (**c**) Opacities in central incisors affected by mild MIH (**d**) Mixed dentition, central incisors with moderate MIH.

MIH is a commonly observed dental condition in children worldwide, characterized by structural defects of the enamel of the first permanent molars and incisors, leading to distinct colored opacities ranging from white to yellow-brown. The global prevalence of MIH shows variable data, with rates between 7% and 11% in Austria, while Germany reports a much higher prevalence of up to 30% [7]. In Romania, a prevalence of 14.3% has been reported [8]. Besides aesthetic concerns, MIH often causes hypersensitivity and an increased predisposition to dental caries [9]. Although MIH is recognized as a global health issue, the specific causes of this condition remain mostly unknown. This condition is classified into four categories: mild (small, well-defined opacities present on one or more surfaces of the affected teeth, but no enamel loss), moderate (larger, more extensive opacities are observed, and there may be some enamel loss or roughness. The enamel surface may start to show signs of wear or slight breakdown), severe (extensive enamel opacities and significant loss of tooth structure are present), and atypical restoration (atypical restorative procedures due to extensive enamel breakdown, inadequate adhesion because of the compromised nature of the underlying tooth structure. Patients presenting with severe demarcated opacities on the enamel, accompanied by immediate post-eruptive breakdown (PEB) of hard dental tissue, are classified as Grade 3 (Figure 2) [10]. Numerous studies exploring potential etiological factors have highlighted the multifactorial nature of this condition linked to prenatal, perinatal, and postnatal factors [11].

The developing tooth bud is sensitive to a wide range of systemic disturbances, and enamel, in particular, cannot regenerate once it is damaged [12,13]. Previous investigations have already shown that both genetic and environmental disruptions during the morphodifferentiation stage can cause abnormalities in tooth shape, size, and structure, with various genes being associated with early tooth morphogenesis [14]. The maturation stage of enamel is representative of the degree of mineralization [15]. Thus, it is necessary to study the influencing factors that act during this stage.

Morphological, numerical, and eruption abnormalities of teeth frequently co-occur, suggesting a possible genetic implication. The literature contains numerous studies investigating potential correlations between MIH and hypodontia [16], as well as other dental or skeletal anomalies such as taurodontism, root dilaceration, anterior open bite, and crossbite [17,18].

Recent studies have identified glucocorticoid receptors in ameloblasts [19]. Glucocorticoids include a variety of hormones that modulate bodily functions, one of the most important being cortisol. Cortisol is a glucocorticoid secreted by the adrenal cortex in response to any stress situation. Cortisol has been implicated in numerous general conditions, including cardiovascular diseases, autoimmune diseases, mental disorders, and even dental conditions [20]. The fact that glucocorticoids have receptors on ameloblasts suggests the need for research into a possible relationship between stress and MIH.

**Figure 3:**
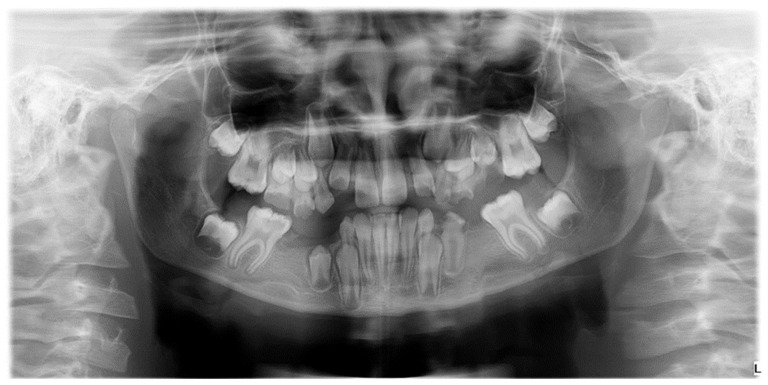
Radiographic image illustrating hypodontia in a patient with mixed dentition and clinical diagnosis of Molar-Incisor Hypomineralization.

Confirming existing evidence regarding etiological links and exploring new, previously undetected associations is of particular importance, given that, to date, no studies have been conducted in Romania on the etiology of MIH in correlation with other coexisting anomalies to optimize treatment. The high prevalence of dental anomalies, especially hypodontia, in children with MIH is a new and clinically significant finding, requiring further research due to its potential implications for evaluation and treatment planning (Figure 3) [21].

The present study is based on the hypothesis that there is no significant correlation between MIH-type enamel development defects and dental number anomalies (hypodontia, supernumerary teeth) but that prenatal, perinatal, and postnatal stress factors have a significant influence on the occurrence of enamel development defects.

### Objectives

The aim of this study is to identify the etiology of enamel developmental defects and to investigate if there is any correlation between MIH-type structural anomalies and numerical anomalies (hypodontia, supernumerary teeth) to optimize interdisciplinary restorative-orthodontic treatment. The secondary objective of this study is to determine the influence of stress factors on the occurrence of enamel developmental defects through a questionnaire that examines the medical history of affected children and their mothers. It investigates significant correlations between relevant aspects of patients’ medical history, prenatal and perinatal conditions, maternal health and habits during pregnancy, early childhood nutrition, and exposure to environmental factors.

## 2. Materials and Methods

This study was conducted between July and September 2024 on an initial sample of 90 patients with mixed dentition, aged between 6 and 11 years, from an urban environment (Transylvania region, Romania), representing all patients who visited the dental office at Natural Smile Dental Clinic by Dr. Pop in Targu-Mures, for various dental care services between June and August 2024. The patients were divided into two equal groups: 45 with MIH and 45 without.

### Patient Selection Criteria

Mixed dentition (6–11 years), originating from the urban environment, with no associated general pathologies and no history of chronic medication. Patients were excluded if their mothers had chronic medication or diseases during pregnancy, if the biological mothers were unable to complete the questionnaire (adopted, foster care, orphans, etc.) if patients had genetic syndromes with dental manifestations (e.g., ectodermal dysplasia, Down syndrome), previous tooth extractions, cleft lip/palate, or fixed orthodontic appliances on the first permanent molars and/or incisors (Table 2).

After applying the exclusion criteria, the final groups included 32 patients in the study group and 25 in the control group. Before the initiation of the study, the informed consent form was distributed and signed by the mothers.

The documentation for these patients included clinical examination, patient’s medical chart, intraoral photographs, digital panoramic radiograph, and a questionnaire completed by the biological mother regarding exposure to certain risk factors during the prenatal, perinatal, and postnatal periods.

The questionnaire distributed to the mothers consisted of 20 questions related to potential etiological factors, divided into the following sections:

- Prenatal Factors:
  ○ Environment (urban, rural)
  ○ Mother’s educational level and age at conception
  ○ Socioeconomic status
  ○ Method of conception (natural or assisted)
  ○ General health conditions during pregnancy, infections, fever, medication, variations in commonly evaluated health indicators (thyroid hormones, calcium, iron, vitamin D), and other pregnancy complications.
- Perinatal Factors:
  ○ Term or premature birth
  ○ Type of delivery (natural or cesarean)
  ○ Duration of labor (prolonged or normal)
  ○ Medication administered to facilitate birth
  ○ Birth weight (low, normal, or high)
  ○ Presence or absence of hypoxia.
- Postnatal Factors:
  ○ General conditions developed by the child in the first months of life
  ○ Type of feeding (breastfeeding, formula)
  ○ Infections
  ○ Variations in usual health indicators (Ca, Mg, Fe, vitamin D)
  ○ Moment of the mother’s return to work.

**Figure 4:**
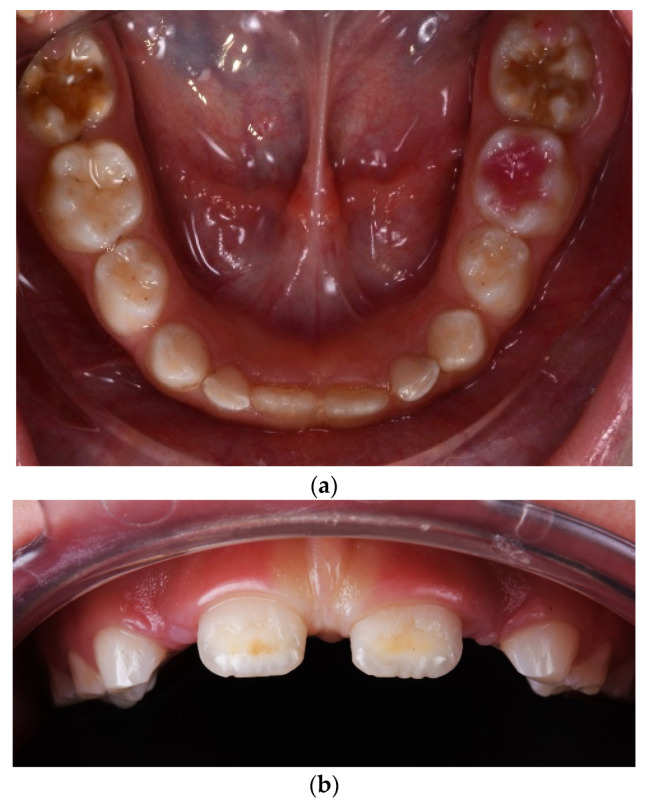
Clinical aspects of enamel developmental defects MIH type. (**a**) Severe structural damage MIH type, grade 3 and posteruptive enamel breakdown (PEB); (**b**) Limited post-eruptive enamel breakdown localized at central incisors.

The clinical diagnosis of MIH was established according to EAAPD criteria [22] following clinical evaluation, and it was documented through intraoral photographs taken in occlusal and frontal views using a Nikon D7500 F18 ISO 200 (Nikon Corporation, Bangkok, Thailand) camera and Doctors Eyes mirrors (Doctorseyes GmbH, Ochsenhausen, Germany) (Figure 4).

Each child was seated in the dental chair with their head supported, and their teeth were examined using dental mirrors and air spray under a standardized light source. The clinical examination was conducted by a pediatric dentist who had been trained prior to the study by an experienced pediatric dentist. Calibration exercises were performed on children who did not participate in the main study.

**Figure 5:**
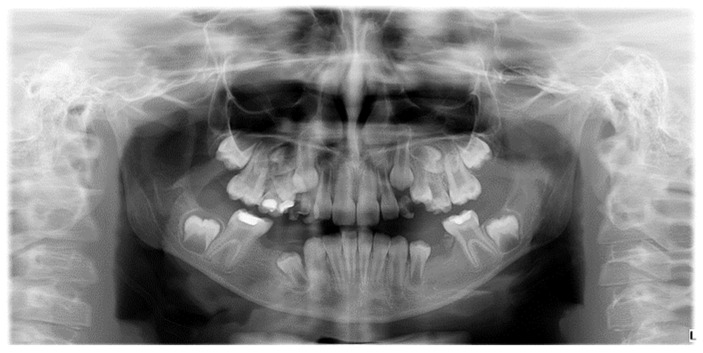
Orthopantomogram of a patient with mixed dentition, clinical diagnosis of MIH, and radiographically certified hypodontia.

Numerical anomalies (agenesis, supernumerary teeth) did not account for the presence or absence of wisdom teeth. The diagnosis was made following clinical examination, anamnesis, and medical history, with dental extractions excluded. Numerical anomalies were confirmed radiographically, based on an orthopantomogram taken with Pax Flex 3D +, Vatech X-ray machine, VATECH Dental Manufacturing Ltd., Sutton, UK, with an exposure time of 12.9 s, 80 kVp, and 9.0 mA (Figure 5).

Questionnaires, as well as data regarding the presence of structural and numerical anomalies, were entered into a database and statistically processed using IBM SPSS software, version 23, Armonk, NY, USA: IBM Corp. The data were treated as nominal or quantitative variables. Nominal variables were characterized using frequencies. A chi-square test was applied to compare the frequencies of nominal variables. Multivariate analysis was conducted using linear regression models. The dependent variable was the group classification (control and case groups). We included as independent variables the data that achieved a significance level of *p* < 0.05 in univariate analysis. The level of statistical significance was set at *p* < 0.05, and the confidence interval was set at 95%.

## 3. Results

The sample of 97 patients included 21 girls and 36 boys, aged between 6.5 and 11 years. The mean age in the study group was 7.5 years (±1.36), and in the control group, it was 7.04 years (±1.09), with no statistically significant difference in age (*p* = 0.17) (Table 1). The mean age of the mothers in the MIH group was 35.56 years (±5.74), whereas, in the control group, mothers were younger, with a mean age of 29.36 years (±3.18), a statistically significant difference (*p* = 0.0001) (Table 3).

In terms of sex distribution, the chi-square test indicated a higher prevalence among boys (66.7%, meaning 24 cases out of 36 boys) compared to girls (38.1%, meaning 8 cases out of 21 girls), with the difference being statistically significant (*p* = 0.036).

Regarding the method of conception, in vitro fertilization was found in 4 cases, 75% of which (3 cases) were in the MIH group, although this was not statistically significant (*p* = 0.37, *p* > 0.05).

Although there were differences between values recorded among mothers in the MIH group compared to the control group concerning other prenatal factors, these differences were not statistically significant. Therefore, the number of pregnancies, as well as potential disruptive factors identified during gestation—such as general conditions (infections, viral illnesses, gestational diabetes, hypertension, hypothyroidism, hemorrhages), administration of medications (antibiotics, non-steroidal anti-inflammatory drugs, hormonal therapies, antispasmodics), variations in thyroid hormones, vitamin D, serum calcium, fever, and other complications (blood group incompatibility, placenta previa, anemia)—did not show statistical significance (Table 4).

Analyzing perinatal factors, obstetrical complications (umbilical cord around the neck, hemorrhages, hypertension), term or preterm birth, birth weight (low, normal, or high), type of delivery (natural or cesarean), and neonatal hypoxia did not demonstrate a statistically significant influence, with *p*-values greater than 0.05.

However, prolonged labor and the administration of medication to facilitate delivery were statistically significant risk factors, with a *p*-value of 0.011 and *p* = 0.003, respectively (Table 5).

Among the postnatal factors, significant differences were identified between the two groups regarding the administration of medication to the child before 12 months (*p* = 0.028), low serum calcium levels (*p* = 0.015), and otorhinolaryngology (ENT) or oral and maxillofacial (OMF) infections (*p* = 0.023). Variations in vitamin D, magnesium, and iron levels, type of feeding (breastfeeding or formula), duration of breastfeeding (up to 3 months or beyond 6 months), and the timing of the mother’s return to work (before or after 6 months) did not show statistically significant differences between the groups (Table 6).

The univariate logistic regression analysis (Table 7) quantified the risk of developing MIH with a 95% confidence interval set as follows:

- Female sex was identified as a protective factor (OR = 0.308).
- Prolonged labor increased the risk by 5.25 times.
- Medications administered to facilitate birth increased the risk of MIH tenfold.
- Medications administered in the first 12 months of life increased the risk fourfold.
- ENT/OMF infections increased the risk by 4.632 times.

**Figure 6:**
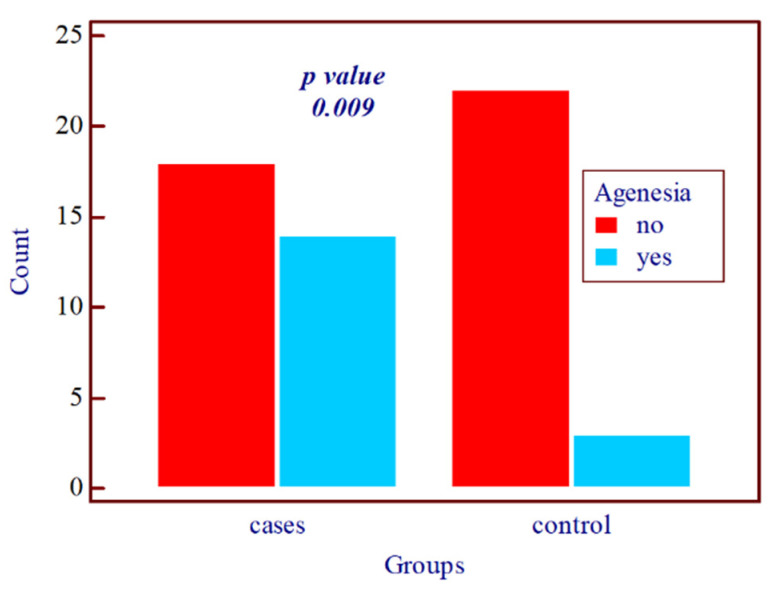
Graphic illustration of agenesis in the study group compared to the control group.

The prevalence of tooth number anomalies (such as agenesia and supernumerary teeth) among the 57 patients was 29.9%, with 82.4% (14 cases) of these anomalies being associated with MIH (molar-incisor hypomineralization). Only 17.6% (3 cases) of the tooth number anomalies were identified in the control group, and the difference was statistically significant, with a *p*-value of 0.009 (Figure 6)

In the control group, two of the three cases of numerical anomalies were identified as supernumerary teeth, while one case was a secondary lower premolar hypodontia found among the 14 total cases of hypodontia.

## 4. Discussion

The permanent first molars begin developing during the fourth month of gestation, with mineralization processes occurring during the perinatal period. While the early maturation phase of the primary molars takes place within the first year of life, the late maturation of the enamel surface extends over several years [23].

Various types of developmental defects can occur in enamel formation, depending on when the activity of ameloblasts is disrupted. Quantitative defects are associated with changes occurring during the matrix deposition/secretion phase, while qualitative defects are linked to disturbances primarily affecting the mineralization process.

Regarding the chronology of these lesions, the localization of hypoplastic enamel defects on the tooth crown has been used to estimate the age of the individual at the time the perturbing factor acted based on general literature data on crown development [24]. Gleiser and Hunt documented the completion of the permanent first molar crowns between 2.5 and 4.4 years of age [25]. More recent studies by Boyde and others have reported the chronological age of enamel completion in the upper central incisor at around 4.64 years, with Reid noting variations based on geographic region regarding the age of crown formation and mineralization [26,27].

Perinatal factors such as prematurity and hypoxia have been associated with enamel defects. This link is justified by the disruption of calcium metabolism in premature infants due to hypoxia resulting from complications of prematurity, which can affect ameloblast function [28].

Regarding the incidence of hypoxia at birth, it was identified more frequently in the MIH group (60%) compared to the control group, with a statistically significant difference (*p* < 0.0001 for the *t*-test). Various studies have explored the correlation between potential stress events, noting that enamel-producing cells have glucocorticoid receptors (e.g., the timing of the mother’s return to work). Among stress-associated events, factors such as weaning and the mother’s return to work were not statistically significant; however, prolonged labor was a risk factor with an Odds Ratio of 5.25. Persistent cortisol responses were observed in separation studies, with higher cortisol levels administered to infants compared to other stress categories [29].

In this study, univariate regression analysis indicated that males are more prone to enamel development defects, with an Odds Ratio of 0.308 and a *p*-value of 0.039. The Wald test, with a value of 4.249 in our study, may sometimes be unreliable, especially when the coefficient is large or the sample size is small. In such cases, the test may give various results, either overstating or understating the significance of a predictor.

Postnatally, changes in health status during the first year of life had a significant correlation with MIH, with a history of illnesses, antibiotic use, and high fever during the postnatal period indicating a fourfold increased risk of developing MIH. All these indicators can be considered stressors, influencing the overall cortisol levels in the body [30]. Variations in cortisol levels during matrix secretion or enamel maturation could contribute to enamel defects, given that corticosteroid receptors have been discovered in rat ameloblasts [19]. A possible correlation between genetic determinism and the modulatory effect of cortisol may be supported by the fact that glucocorticoids modulate *Tgfbr3*. Intraperitoneal administration of dexamethasone (10 mg/kg) for 24 h in alive mice increased the abundance of mRNA for *Tgfbr3*. The relationship between *TGFBR1* gene activation and the development of MIH was also investigated, as recent studies suggest a potential association [31,32].

Significant correlations for cortisol levels at bedtime were observed in breastfeeding mother-infant pairs, while formula-fed pairs did not show these correlations. This data suggests that multiple factors may contribute to the observed cortisol synchronization in mother-infant pairs, including the transfer of cortisol through breast milk [33]. However, in this study, these events did not show statistical significance (*p*-value > 0.05).

Existing meta-analyses suggest that the likelihood of developing MIH is associated with the mother’s health status. Children whose mothers experienced health problems during pregnancy had a fourfold increased risk of developing MIH [34]. Therefore, our study also investigated potential general conditions during pregnancy (such as gestational diabetes, pregnancy-related hypothyroidism, bacterial, viral, or fungal infections, hypertension, dyslipidemias, and abnormal levels of calcium, magnesium, iron, zinc, and vitamin D). However, except for vitamin D deficiency (*p* > 0.005), no identified pathology had statistical significance. Previous research indicates that neither mineral supplementation nor a daily dose of 1000 IU of vitamin D, compared to a lower dose of 500 IU, reduced the prevalence of enamel defects in primary or permanent dentition [35]. Studying electrolyte imbalances (Ca, Mg, Fe, Zn) is important in patients with MIH (molar-incisor hypomineralization), as amelogenesis follows a well-defined sequence of cellular proliferation, differentiation, maturation, and apoptosis, characterized by specific gene expression patterns. Ameloblasts sequentially secrete enamel matrix proteins (amelogenin, enamelin, ameloblastin) and proteases (KLK4 and MMP20). These proteases degrade the enamel matrix, allowing further growth of mineral crystals under optimal pH and ionic conditions [36].

Bones and teeth, as primary mineralized tissues, are regulated by many of the same genes and hormones [37]. Parathyroid hormone-related protein acts to delay terminal differentiation in developing endochondral bone chondrocytes and forming teeth. Both bones and teeth are similarly affected by systemic disorders of mineral homeostasis or extracellular matrix. Unlike bones, which can regenerate through remodeling, teeth do not remodel once formed, making the effects of systemic disorders permanent [38].

It is also well known that pathological variations in vitamin D correlate with the presence of hypodontia in certain syndromes [39,40].

Hypodontia may not exist as a singular entity. Other dental anomalies related to size and shape, such as double teeth, microdontia, and taurodontism, may coexist [41].

Populations exposed to high levels of malnutrition and disease, from prehistoric times to the present, exhibit elevated rates of linear enamel hypoplasia. Although these defects appear to be associated with episodes of malnutrition and infection, their specific etiology remains unknown [42]. Moreover, some studies dispute the correlation between diet and the development of MIH-type enamel defects.

Systematic reviews have concluded that most environmental factors previously studied and considered risk factors for MIH (molar-incisor hypomineralization) do not fully explain the disease’s etiology [43,44]. In contrast, etiological factors associated with enamel-forming genes or genes related to immune response have been reported to play a role in the onset of this condition [45].

From a clinical perspective, it is important to recognize the potential for multiple oral/dental anomalies to be present in the same patient. Recent recognition has been given to the potential for additional anomalies in children with MIH [46]. An observational study of 101 children with MIH conducted at a dental hospital in the UK showed that 29% of patients had an additional dental anomaly. A key finding was a high prevalence of hypodontia, affecting 12% of MIH patients (16.1% of girls and 2.6% of boys). These findings have sparked interest within the international pediatric dentistry community [21].

Ultimately, tooth extraction within the framework of orthodontic treatment should be considered as an alternative to avoid a cycle of failure and multiple dental procedures in anxious children. The first clinical step in severe MIH cases is to decide whether to restore or extract these permanent first molars, considering factors such as the child’s age, level of understanding and cooperation, degree of dental crowding, type of malocclusion, presence of agenesis, wisdom teeth, condition of adjacent teeth, long-term prognosis of restorations, and the need for endodontic treatment in case of irreversible pulp inflammation. Treatment of MIH grade 3 lesions with PEB (preformed metal crowns) is complex and requires close and early collaboration between the pediatric dentist and orthodontist to determine the optimal timing for molar extraction. The ideal age should be radiographically indicated by the onset of calcification of the bifurcation of the lower second molar (maxilla 8.5–10 years, mandible 10.5–11.5 years) [47,48].

Understanding the prevalence and characteristics of MIH has significant implications for improving the quality of life of affected individuals, as dental conditions are closely related to patients’ mental well-being [49]. It is essential to understand the complexity of MIH not only for therapeutic management but also to develop preventive strategies and public health education programs. Prevalence and characteristics of MIH are key factors in elucidating the complexity of this condition. Gaining this understanding is crucial for developing effective public health programs, targeted treatments, and educational initiatives. Identifying the etiological factors is not merely an academic exercise but essential for advancing oral health. With the right policies, treatments, and educational strategies, it is possible to influence the prevalence of MIH.

Limitations of the Study: The small sample size of 57 participants limits the generalizability of the findings, and a larger sample size would provide more robust data. The geographic limitation of the urban setting may reduce the applicability of results to children in rural or different socio-economic environments, where risk factors such as nutritional deficiencies and healthcare access could influence MIH prevalence and severity. Also, the reliance on maternal self-reporting through questionnaires could introduce recall bias, particularly for events during pregnancy and labor, which may affect the accuracy of identifying the etiology of MIH and its co-occurrence with hypodontia.

Given the observed correlation between MIH and hypodontia in the current study, it is possible that certain genetic factors contribute to the occurrence of both conditions. Identifying these genetic markers in a further study could help in early diagnosis and personalized treatment planning. A prospective cohort study is needed, recruiting women planning to conceive in the future to enable the assessment of risk factors from the preconception period through pregnancy and up to one year post-birth in order to clarify the co-occurrence of MIH and other dental anomalies.

## 5. Conclusions

- The study highlights a significant association between Molar Incisor Hypomineralization (MIH) and specific prenatal, perinatal, and postnatal factors, such as advanced maternal age and complications during labor. The observed correlation between MIH and various dental anomalies underscores the necessity for an interdisciplinary approach in both diagnosis and treatment.
- The study reveals a strong correlation between Molar Incisor Hypomineralization (MIH) and dental agenesis, suggesting that children with MIH are at a higher risk of missing teeth. This significant association emphasizes the need for proactive management strategies in patients with MIH, where early identification of concurrent dental agenesis can inform more effective, individualized treatment plans. Integrating orthodontic and restorative interventions early in the management process is crucial to address both MIH and the related dental anomalies, ultimately improving long-term oral health outcomes for affected children.

## Figures and Tables

**Table 1:** Developmental Dental Anomalies (DDA)-simplified classification.

Type of Dental Developmental Anomaly (DDA)	Number	Size	Shape	Structure
Clinical Manifestation	Anodontia Oligodontia *Hypodontia* *Supranumerary teeth* Pleiodontia	Macrodontia Microdontia	Fusion Gemination Peg-shape Dens in dente Dens invaginatus Enamel pearl Taurodntism	Hypoplasia Hypomieralization *Molar-Incisor Hypomineralization* Fluorosis Amelogenesis imperfecta Dentinogenesis imperfecta

**Table 2:** Patient selection criteria.

Inclusion Criteria	Exclusion Criteria
Children with mixed dentition	Biological mother unable to complete questionnaire (adopted, orphans, foster care)
Age between 6–11 years old	Mothers with chronic medication during pregnancy
Urban environment	No previous tooth extractions
No general diseases or syndromes	Patients with genetic syndromes with dental manifestation (Down Syndrome, ectodermal dysplasia) Cleft lip/palate
No history of chronic medication	Orthodontic fixed appliances on the first permanent molars/incisors

**Table 3:** The mean age of participants and their mothers, with Standard Deviation (SD).

	Patient’s Mean Age (Years) with SD	Mother’s Mean Age (Years) with SD
Case	7.50 ± 1.36	35.56 ± 5.74
Control	7.06 ± 1.09	29.36 ± 3.18
*p*-value	0.17	0.0001

**Table 4:** Prenatal factors and their statistical significance.

No Crt	Prenatal Factor	*p*-Value
1	Natural conception or in vitro fertilization	0.37
2	Number of pregnancies	0.60
3	General diseases during pregnancy	0.22
4	Medication during pregnancy	0.14
5	Variation of thyroid hormones	0.95
6	Variation of Vitamin D serum level	0.85
7	Variation of Calcium serum level	0.38
8	Infections during pregnancy	0.28
9	Fever during pregnancy	0.26
10	Gestational complications	0.58
11	Term or preterm birth	0.94

**Table 5:** Perinatal risk factors identified in our study.

	Labor Induction Medication	Prolonged Labor
Cases (%)	93.8%	50%
Control (%)	40%	16%
*p*-value	0.003	0.011

**Table 6:** Postnatal factors and their statistical significance.

Postnatal Factor	Percentage in Case Group	Percentage in Control Group	*p*-Value	Statistically Significance
Hypoxia at birth	18.8%	12.4%	0.122	No
General diseases in first months	56.1%	43.9%	0.57	No
*Medication in first months after birth*	*50%*	*20%*	*0.028*	*Yes*
Low levels of Vitamin D in serum	9.4%	4%	0.62	No
*Low levels of Calcium in serum*	*21.9%*	*0%*	*0.015*	*Yes*
Low levels of Magnesium in serum	0%	0%	-	-
Low levels of Iron in serum	28%	16%	0.35	No
Breastfeeding less than four months	51.6%	48.4%	0.59	No
Formula before six months	60.9%	39.1%	0.59	No
*ENT/OMF infections in first year*	*16.9%*	*16%*	*0.023*	*Yes*
Mother’s return to work before six months	40.6%	36%	0.72	No

**Table 7:** The univariate logistic regression analysis.

	B (Regression Coefficient)	SE (Standard Error)	Wald	*p*-Value	O.R. (Odd Ratio)
Female sex	−1.179	0.572	4.249	0.039	0.308
Prolonged labor	1.658	0.650	6.506	0.011	5.250
Medication to facilitate birth	2.303	0.837	7.544	0.006	10.000
Medication in first twelve months	1.386	0.612	5.125	0.024	4.000
ENT/OMF infections	1.533	0.650	5.555	0.180	4.632

## Data Availability

The original contributions presented in the study are included in the article, further inquiries can be directed to the corresponding author.
